# Andean Plants Essential Oils: A Scented Alternative to Synthetic Insecticides for the Control of Blowflies

**DOI:** 10.3390/insects12100894

**Published:** 2021-10-01

**Authors:** Priscilla Farina, Francesca Venturi, Roberta Ascrizzi, Guido Flamini, Rodrigo Daniel Chiriboga Ortega, Maria Cristina Echeverría, Sania Ortega, Angela Zinnai, Stefano Bedini, Barbara Conti

**Affiliations:** 1 Department of Agriculture, Food and Environment, University of Pisa, Via del Borghetto 80, 56126 Pisa, Italy; priscilla.farina@phd.unipi.it (P.F.); francesca.venturi@unipi.it (F.V.); angela.zinnai@unipi.it (A.Z.); barbara.conti@unipi.it (B.C.); 2 Department of Pharmacy, University of Pisa, Via Bonanno 6, 56126 Pisa, Italy; roberta.ascrizzi@gmail.com (R.A.); guido.flamini@unipi.it (G.F.); 3 Department of Biotechnology, Universidad Técnica del Norte, Av. 17 de Julio, 5-21 y Gral, José María Cordova, Ibarra 100105, Ecuador; rdchiribogao@utn.edu.ec (R.D.C.O.); mecheverria@utn.edu.ec (M.C.E.); smortega@utn.edu.ec (S.O.)

**Keywords:** botanical insecticides, repellents, sensory analysis, Andean flora, *Aloysia citrodora*, *Bursera graveolens*

## Abstract

**Simple Summary:**

Blowflies play a key role in the transmission of foodborne diseases and cause myiasis. With their repellent and insecticidal properties, essential oils (EOs) from aromatic plants can control such insects. However, because of their strong odour, they are often unsuitable for protecting food or places. In this work, the EOs of two Andean plants, *Aloysia citrodora* and *Bursera graveolens*, known for their pleasant odour, were analysed from a chemical and sensory point of view, and their bioactivity against the blowfly *Calliphora vomitoria* was assessed in comparison with the highly effective, but bad-smelling, *Allium sativum* EO. The behavioural test showed that the *A. citrodora* EO was more repellent than the *A. sativum* EO and that, on the contrary, the *B. graveolens* EO was attractive to *C. vomitoria.* The toxicity tests showed that the EOs of both Andean plants have a clear insecticidal effect against blowfly eggs and adults. In terms of contact with adult flies, the *A. citrodora* EO was about twice as toxic as the *A. sativum* EO.

**Abstract:**

Blowflies are vectors of microorganisms and human pathogens, and their maggots cause myiasis in vertebrates and infest and spoil meat and fish products. Essential oils (EOs) from spices were proven to be a safer and more sustainable alternative to synthetic insecticides for the control of blowflies and are suitable for protecting food from such pests. However, some EOs are not acceptable for environmental or topical applications due to their strong, unpleasant odour. In this study, we measured the acute toxicity and the repellence of two EOs extracted from the Andean plants *Aloysia citrodora* Palau and *Bursera graveolens* (Kunth) Triana and Planch., both known for their pleasant odour, against the blue blowfly *Calliphora vomitoria* (L.) (Diptera: Calliphoridae). We also compared their bioactivity with that of the *Allium sativum* L. EO, which is very effective but bad-smelling. The *A. citrodora* EO was mainly rich in oxygenated monoterpenes, the most abundant of which were geranial (26.8%) and neral (21.0%). The *B. graveolens* EO was chiefly composed of monoterpene hydrocarbons, mostly represented by limonene (46.2%). According to the sensory description, the best odour profile was associated with the *A. citrodora* EO, while the olfactory expression of the EO from *B. graveolens* was negatively affected by a strong odour of “mouldy lemon”. The behavioural test showed that the *A. citrodora* EO was more repellent than that of *A. sativum* and, on the contrary, that the *B. graveolens* EO was attractive. The toxicity tests showed that the EOs of both Andean plants have a clear toxic effect on the *C. vomitoria* eggs and adults. In terms of ovicidal activity, there were no significant differences among the effects of the three tested EOs. On the adult flies, the toxicity of the two EOs of the Andean plants was much lower than that of *A. sativum* (LC_50_ fumigation = 1.86 μL EO L^−1^ air; LC_50_ ingestion = 8.10 μL EO mL^−1^ gel) both by fumigation (LC_50_ = 23.66 and 25.30 μL EO L^−1^ air for *A. citrodora* and *B. graveolens,* respectively) and ingestion (LC_50_ = 36.65 and 44.97 μL EO mL^−1^ gel for *A. citrodora* and *B. graveolens,* respectively), while, by contact, the *A. citrodora* EO (LD_50_ = 0.27 μL EO insect^−1^) was more toxic than the *A. sativum* EO (LD_50_ = 0.46 μL EO insect^−1^).

## 1. Introduction

Blowflies (Diptera: Calliphoridae) play a key ecological role in decomposing carrions [1,2,3,4,5] and in pollination [6,7,8]. However, because of their feeding and reproductive behaviour, blowflies are also vectors of microorganisms and human pathogens [9,10,11], which they spread on food and surfaces as they land on them [11,12]. In addition, blowfly maggots cause myiasis and infest and spoil products in slaughterhouses, meat and fish industries, and stores [13,14,15,16].

Currently, the control of blowflies mainly relies on the massive use of broad-spectrum neurotoxic insecticides (e.g., organophosphate, pyrethroids, and spinosad) and insect growth regulators (e.g., cyromazine, dicyclanil, and diflubenzuron) [17,18,19]. The extensive use of such insecticides has led to the development of resistance to one or more (cross-resistance) of the synthetic compounds used [20,21] and to negative effects on the environment and on human and animal health [22,23,24].

A safer and more sustainable alternative to the use of synthetic insecticides for the control of blowflies are essential oils (EOs) [16,25,26,27,28]. Although the effectiveness of several EOs was widely proven, they are not so widely applied yet, as it would be expected in real life. One of the reasons for such limited use is certainly their distinctive odour. In previous works, we tested the susceptibility of *Calliphora vomitoria* (L.) (Diptera: Calliphoridae) to the toxic activity of several EOs distilled from aromatic plants traditionally used as culinary herbs [25,27,28]. The EO from *Allium sativum* L. (Amaryllidaceae) was the most effective one, and it was successfully used as a component of an emulsion, which was sprayed as a mist to build an olfactory barrier and discourage blowflies from entering a meat-processing room of a ham-curing factory [27]. However, the strong and unpleasant odour of the *A. sativum* EO makes it unsuitable for places other than a factory. For these reasons, in this study we tested EOs with odours that could be compatible with public and private places visited by people, such as hospitals, houses, shops, restaurants, etc. Hence, we focused on two EOs extracted from Ecuadorian plants, *Aloysia citrodora* Palau (Verbenaceae) and *Bursera graveolens* (Kunth) Triana and Planchon (Burseraceae), both known for their pleasant odour.

*A. citrodora* is a perennial shrub native to South America, popularly known as lemon verbena or, in Spanish, cedrón [29]. It is commonly used in folk medicine to treat fever, cold, asthma, headache, spasms, type 2 diabetes, anxiety, and insomnia as well as a diuretic, stomachic, tonic, carminative, and sedative [30,31,32,33]. Alongside its pharmaceutical value, it is used as a flavouring agent in the food and beverage industry for its lemony scent that is a perfect match for fruits and seafood dishes [34,35].

*B. graveolens* is a deciduous tree commonly known as palo santo (“holy wood”). The species is distributed throughout the dry forests, from southern Mexico to north-western Peru [36]. Its woody material has a characteristic spicy, sweet, and balsamic odour and is used as a type of incense [37]. In Ecuadorian and Peruvian traditional medicine, *B. graveolens* is employed against flu, asthma, dermatitis, stomach ache, inflammatory diseases, and rheumatisms [30,31,38].

The aim of this study was to determine the chemical composition of the *A. citrodora* and *B. graveolens* EOs, to assess their olfactory profiles, and to test their toxicity and repellence against *C. vomitoria* in order to select potential good-smelling EOs for the control of blowflies.

## 2. Materials and Methods

### 2.1. Plant Material

*A. citrodora* leaves and *B. graveolens* stems were collected in the Intag Valley (0°18′15″ N, 78°34′27″ W), which is part of the northern Andes, in the province of Imbabura, Ecuador. These species are part of an agroforestry system located at 900 to 1200 m above sea level, with an annual rainfall of 1500–1750 cc^3^ and an average temperature of 20 to 22 °C.

Due to its strong repellence and toxicity against Calliphoridae [27], the *A. sativum* EO was included in the trials as the positive control EO. This EO was purchased from Vis Medicatrix Naturae s.r.l. (Florence, FI, Italy), and stored in glass vials at 4 °C until use.

### 2.2. Extraction and Chemical Analyses of the Essential Oils

The extraction of the *A. citrodora* and *B. graveolens* EOs was conducted at the Department of Biotechnology, Universidad Técnica del Norte, Ibarra (Ecuador). The plants were air-dried, and the EOs were obtained by steam distillation, using a Clevenger system for 3 h. The resulting EOs were dried over anhydrous sodium sulphate and stored in glass vials at 4 °C until use.

The chemical analyses were conducted at the Department of Pharmacy, University of Pisa, Italy. The hydrodistilled EOs were diluted to 0.5% in HPLC-grade *n*-hexane and then injected into a gas chromatography-electron impact mass spectrometry (GC-EIMS) instrument. GC-EIMS analyses were performed using a Varian CP-3800 gas chromatograph (Agilent Technologies Inc., Santa Clara, CA, USA), equipped with an HP-5 capillary column (30 m × 0.25 mm, coating thickness 0.25 μm) and a Varian Saturn 2000 ion trap mass detector (Agilent Technologies Inc., Santa Clara, CA, USA). The analytical conditions were as follows: the injector and transfer line had temperatures of 220 and 240 °C, respectively; the oven temperature was programmed at 60 to 240 °C, at 3 °C/min; the carrier gas was helium at 1 mL/min flow rate; the injection volume was 1 μL (0.5% HPLC-grade *n*-hexane solution); the split ratio was 1:25. Data acquisition included: full scan; scan range: 30–300 *m/z*; scan time: 1.0 s.

The identification of the constituents was based on a comparison between their retention times and those of the original samples, by comparing their linear retention indices relative to the series of *n*-hydrocarbons. Computer matching was also used to search commercial [39] and laboratory-developed mass spectral libraries built up from pure substances and components of commercial EOs of known composition and MS literature data [40].

The chemical composition of the *A. sativum* EO, analysed with the same methods as described above, was reported in Bedini et al. [27].

### 2.3. Sensory Analysis of the Essential Oils

The odour profiles of the *A. citrodora*, *B. graveolens*, and *A. sativum* EOs were evaluated by a trained panel of eight assessors (“expert panel” of the Department of Agriculture, Food and Environment, University of Pisa) [41,42]. All assessors had previous experience in descriptive sensory analyses and were provided with a specifically developed sensory sheet consisting of an unstructured, descriptive parametric score chart. The panellists described the main odours of each sample based on defined odour descriptors such as “intensity”, “persistency”, and “pleasantness” as hedonic parameters. To give a quantitative measure (score) of each descriptor, the panellists were asked to refer to a continuous scale of 0 (minimum level) to 10 (maximum level). Furthermore, the assessors were also asked to provide a list of specific olfactory descriptors of their choice, describing the olfactory profiles of the tested EOs.

The blind odour test was performed in the morning, in a well-ventilated, quiet room and in a relaxed atmosphere. Each panellist was provided with a fragrance tester strip soaked in 10 μL of an unknown EO. To avoid cross-contamination, the three samples were separately assessed in the same morning (with a 15 min break between assessments).

### 2.4. Rearing of Calliphora Vomitoria

*Calliphora vomitoria* were reared according to Bedini et al. [25,27,28], with minor changes. Larvae of *C. vomitoria* were purchased from the retailer Altomare (Vittoria Apuana, LU, Italy) and reared under laboratory conditions (23 °C, 60–70% RH, natural photoperiod). The larvae were fed beef mince, until they pupated. The identification of the species was performed on the emerged adults. Adult flies were put in a 75.0 × 75.0 × 115.0 cm knitted mesh and polyester cage (BugDorm-2400 Insect Rearing Tent, MegaView Science Co., Ltd., Taichung, Taiwan) and kept under laboratory conditions. The flies were fed water and sucrose mixed with yeast (20% *w*:*w*) *ad libitum*, to provide the appropriate amount of protein to stimulate oviposition [43,44].

### 2.5. Behavioural Assay

The repellence or attractiveness of the *A. citrodora*, *B. graveolens*, and *A. sativum* EOs was evaluated in a two-way olfactometer, composed of a cylindrical Plexiglas tube (9.0 cm diameter × 60 cm length) connected by two PVC elbow pipes (2.0 cm diameter × 15.0 cm total length) to two lateral glass chambers (800 mL volume). An opening (10.0 × 5.0 cm) in the central tube was covered with a net for ventilation, and the flies’ entrance on the top was closed with a cap. The collecting chambers on the sides were covered with a black plastic tarp to prevent light influencing the flies and were provided with water and sucrose *ad libitum*. In the collecting chambers, 100 μL of 0.0 (control), 0.05, 0.10, 0.50, 1.0, and 2.0% ethanol (EtOH) solutions of the three EOs (corresponding to 0.0, 0.06, 0.12, 0.62, 1.25, and 2.50 µL EO L^−1^ air) were poured on a square (3.5 × 3.5 cm) of filter paper. Before using it, the solvent was made to evaporate from the paper under a vertical fume hood for 3 min. Groups of five unsexed adult flies (10–15 days old) were released in the central tube through the entrance. After 24 h, the number of flies in the control chamber (NT) or in the EO-treated chamber (T) was counted. Each concentration of the EOs was tested five to twenty times (replicates).

### 2.6. Toxicity Bioassays

The toxicity to the *C. vomitoria* eggs was tested according to Bedini et al. [25,27,28], with minor changes. Adult females were supplied with warm beef mince to stimulate oviposition of the necessary eggs. Squares of filter paper (4.5 × 4.5 cm, area 20.25 cm^2^) were put in glass Petri dishes (10 cm diameter) and treated with 100 μL of 0.0 (control), 0.125, 0.25, 0.50, 0.75, 1.0, and 1.25% EtOH solutions of the EOs (corresponding to 0.0, 0.006, 0.012, 0.024, 0.037, 0.049, and 0.061 µL EO cm^−2^ of filter paper). After the solvent had evaporated from the paper for 3 min under a vertical fume hood, the paper was moistened with 380 μL of water, and 50 eggs (1–3 h old) were placed on the treated part of the paper using a wet brush. The Petri dishes containing the eggs, sealed with Parafilm™, were then incubated at 27 °C in the dark, in a climatic chamber (KW Srl., Siena, SI, Italy). Each concentration of the EOs was tested five times (five replicates). The empty chorions of the hatched eggs were counted daily for 72 h, with the help of a stereomicroscope (Nikon SMZ1500, Nikon Instruments Inc., Tokyo, Japan). At each daily check, the filter paper was wetted again with 380 µL of water.

For the assessment of the EOs toxicity by fumigation, groups of 10 unsexed adult flies (10–15 days old) were put in cylindrical glass chambers (330 mL volume) and provided with water and sucrose *ad libitum*. The chambers were closed with screw lids (6.5 cm diameter). Under the lid, 0.0 (control), 2.0, 4.0, 6.0, 8.0, 10.0, and 12.0 μL of the EOs (corresponding to 0.0, 6.66, 13.33, 20.0, 26.66, 33.33, 40.0 µL EO L^−1^ air) were dispensed on a square (3.5 × 3.5 cm) of filter paper. To avoid any direct contact between the *C. vomitoria* flies and the EO, a cotton gauze was placed between the chamber and the lid and secured with a rubber elastic band. The lid was removed after 24 h of treatment, and the flies’ mortality was checked after 1 h to let the knocked down specimens recover. Then, all the flies were moved into clean Plexiglas cages (15 cm diameter × 14 cm length) with a knitted mesh opening at the back for ventilation and provided with water and sucrose *ad libitum*. Mortality was checked again after another 24 h (48 h after the beginning of the fumigation assay). Each concentration of the EOs was tested four times (four replicates).

To measure the toxicity by contact, adult flies (10–15 days old) were treated with a topical application of different doses of the EOs, using a Burkard micro-applicator (Burkard Scientific Ltd., Uxbridge, United Kingdom) equipped with a 1 mL syringe. The flies were treated with 2 μL of 0.0 (control), 5.0, 10.0, 15.0, 20.0, 30.0, 40.0, 50.0, and 60.0% of EtOH solutions of the EOs (corresponding to 0.0, 0.10, 0.20, 0.30, 0.40, 0.60, 0.80, 1.0, 1.20 µL EO fly^−1^) applied on the thorax of 20 specimens per EO concentration. Each concentration of the EOs was tested three times (three replicates). To ease the application of the solutions, the flies were put in a Falcon tube with a netted cap and anesthetised at −18 °C for 3 min. The treated insects were then kept in Plexiglas cages (20 cm diameter × 30 cm length) with a knitted mesh opening at the back for ventilation and fed sugar and water *ad libitum*. The flies’ mortality was checked after 48 h. The procedure was carried out according to Bedini et al. [26,27,28], with minor changes.

The toxicity by ingestion was assessed in groups of 10 unsexed adult flies (10–15 days old) kept in Plexiglas cages (15 cm diameter × 14 cm length) with a knitted mesh opening at the back for ventilation and fed on water *ad libitum* and 2 mL of a gel containing 0.0 (control), 0.25, 0.50, 1.0, 1.50, 2.50, 5.0, 6.0, 7.5, 10.0, and 15.0% (*w*:*v*) of the EOs (corresponding to 0.0, 2.5, 5.0, 10.0, 15.0, 25.0, 50.0, 60.0, 75.0, 100.0, and 150.0 µL EO mL^−1^ gel). The gel was made by mixing water, sucrose (12.5%), and agarose (0.5%) on a hot plate stirrer (VELP Scientifica, Usmate, MB, Italy), at 125 °C and 500 rpm for 25 min. A total of 2 mL of the gel was left to cool down in Bakelite caps, then incorporated with different concentrations of the EOs. The caps were covered with a square (2.5 × 2.5 cm) of cotton gauze to prevent the *C. vomitoria* flies from drowning while feeding themselves. The flies’ mortality was checked after 48 h.

All mortality rates were corrected using Abbott’s formula [45].

### 2.7. Data Analysis

The reliability of the sensory data collected during the panel test was evaluated by Big Sensory Soft (BSS^®^) version 2.0, a software specifically developed by the Centro Studi Assaggiatori (Brescia, BS, Italy) to process sensory data from panel tests. Data were processed through the Kruskal-Wallis test, with the score for the hedonic parameters as test variables and the EO as a grouping factor. Medians were separated by Dunn-Bonferroni pairwise comparisons.

The proportion of individuals choosing the EO-treated chamber in the two-choice behavioural assays were compared by means of a likelihood-ratio chi-square test, with a null hypothesis of a 50:50 chance of insects choosing the control chamber (NT) vs. the EO-treated chamber (T).

The relative toxicity of the EOs was assessed, using probit analysis [46,47], by calculating the median lethal concentration (LC_50_) for the ingestion and fumigation tests and the median lethal dose (LD_50_) for the contact test. For each toxicity test, a probit model was built for the three EOs. The fitness of the probit model [PROBIT(p) = Intercept + BX; where PROBIT(p) is the cumulative probability estimates, B is the slope of the model, and X is the EO concentration/dose transformed using the base 10 logarithm (covariate)] was tested through the Pearson goodness-of-fit test. A heterogeneity factor was used in the calculation of confidence limits when the significance level was less than 0.150. Differences between LC/LD_50_ values for the three EOs were assessed by relative median potency (rmp) estimates. Differences were considered significant if the rmp 95% confidence interval did not include 1. Statistical analyses were performed via SPSS 22.0 software (IBM SPSS Statistics, Armonk, North Castle, New York, NY, USA).

## 3. Results

### 3.1. Chemical Composition of the Essential Oils

The compositions of the three EOs are reported in Table 1. The EOs analysis identified 40 compounds in the *A. citrodora* EO and 23 in the *B. graveolens* EO, corresponding to 98.6 and 95.7% of their total composition, respectively. The main components were geranial (26.8%), neral (21.0%), and limonene (7.2%) in the *A. citrodora* EO; limonene (46.2%), and α-terpineol (17.8%) in the *B. graveolens* EO.

The chemical composition of the *A. sativum* EO (Table 1) was already investigated and reported in Bedini et al. [27]. Its analysis identified a total of 27 compounds, corresponding to 94.8% of the total composition. The main components were sulfur compounds: diallyl trisulfide (23.1%), diallyl tetrasulfide (17.4%), and diallyl disulfide (16.1%).

### 3.2. Sensory Profiles of the Essential Oils

**Figure 1:**
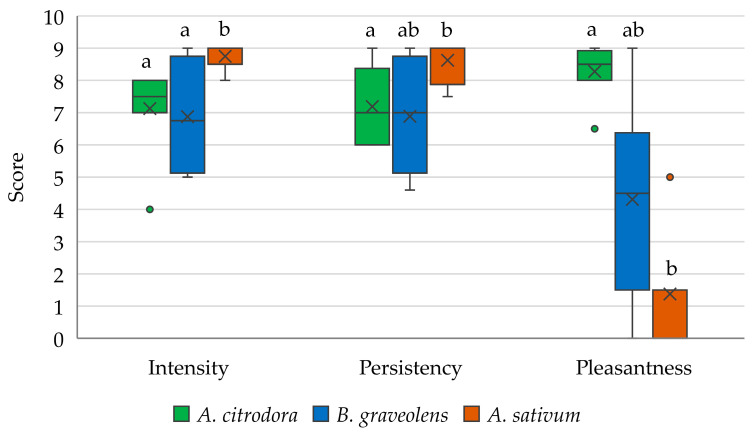
Scores attributed by the expert panel to the main hedonic parameters describing the *Aloysia citrodora, Bursera graveolens*, and *Allium sativum* essential oils (EOs). The box plots show the distribution of scores from the 25th to the 75th percentile. In each box, the middle line represents the median value, the x represents the mean value, the upper and lower vertical lines represent the maximum and minimum values, respectively, and the filled circles represent the outliers. Different letters indicate significant differences according to Dunn-Bonferroni pairwise comparisons test (*p* ≤ 0.05).

The results of the main hedonic parameters measured by the sensory analysis of the three EOs are reported in Figure 1.

The EOs differed significantly in all of the analysed hedonic parameters (*χ*^2^ = 10.1, *p* = 0.06; *χ*^2^ = 7.4, *p* = 0.025; *χ*^2^ = 14.5, *p* = 0.001 for intensity, persistence, and pleasantness, respectively). The *A. sativum* EO was characterised by the highest odour intensity and persistence, together with the lowest pleasantness. The EOs from *A. citrodora* and *B. graveolens* showed the same odour intensity and persistence, but the highest pleasantness was attributed to the former.

**Figure 2:**
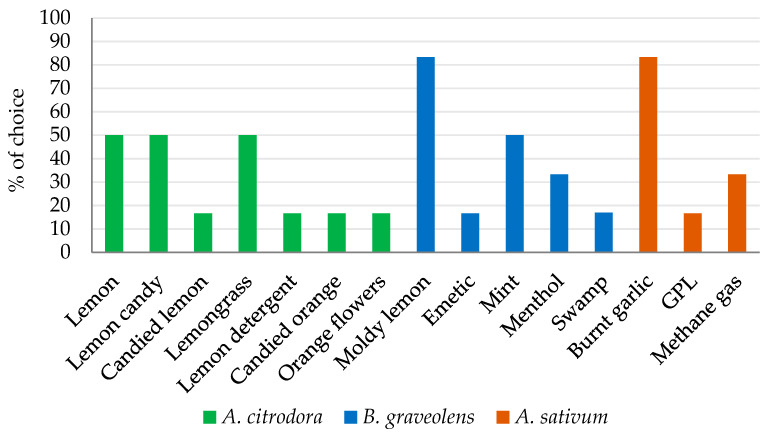
Specific olfactory descriptors and the percentage of times they were used by the expert panel (*n* = 8) for characterising the odours of *Aloysia citrodora, Bursera graveolens*, and *Allium sativum* essential oils (EOs).

Figure 2 lists the specific descriptors used by the eight panellists to describe the three EOs during the olfactory tests, together with their percentage of choice. The three tested EOs showed very different olfactory profiles. Among them, the smell of the *A. citrodora* EO was described by all panel experts as citrusy and floral, thus indicating its high odour complexity. The very low pleasantness score attributed to the *A. sativum* EO can be easily explained by the high number of off-flavours mentioned by the panellists to describe its smell.

### 3.3. Behavioral Response of the C. vomitoria Adults to the Essential Oils

**Figure 3:**
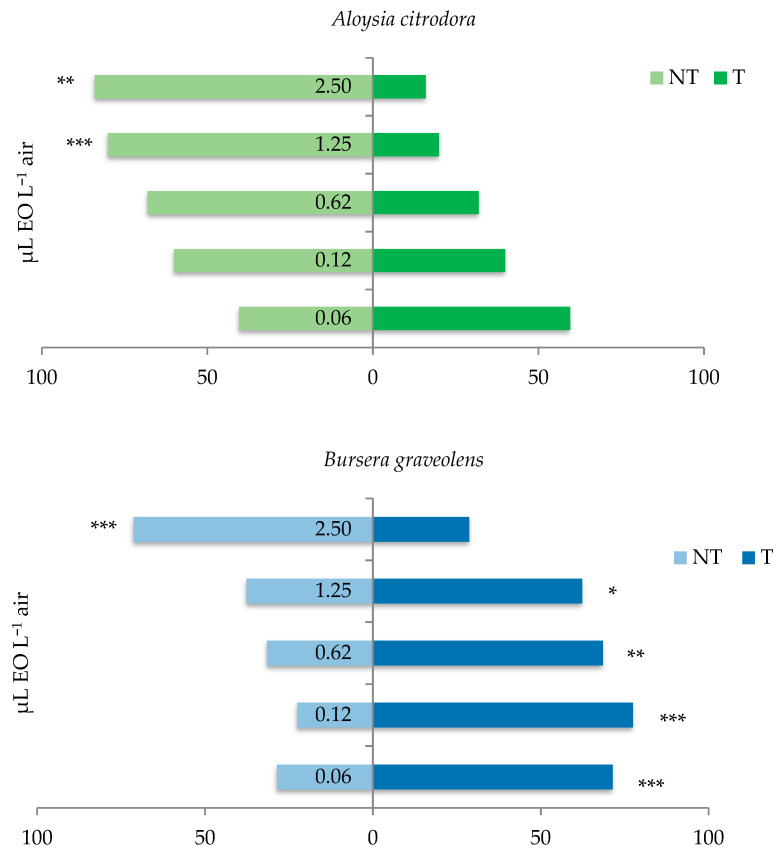

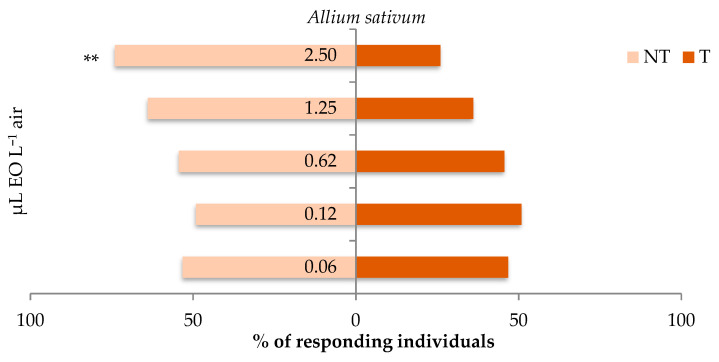
Behavioural response of the *C. vomitoria* adults in the presence of *Aloysia citrodora, Bursera graveolens*, and *Allium sativum* essential oils. NT, % of insects that chose the control chamber; T, % of insects that chose the EO-treated chamber. Asterisks indicate the significant effect of the EOs on the ratio of the adults choosing either the control or the EO-treated chamber (*χ*^2^ test; * *p* < 0.05; ** *p* < 0.01; *** *p* < 0.001).

The two-choice assays proved that the EOs had different effects on the adults of *C. vomitoria*. At the tested concentrations, the *B. graveolens* EO showed an overall positive chemotaxis with significant attractiveness from 0.06 to 1.25 µL EO L^−1^ air (0.06 µL EO L^−1^ air: *χ*^2^ = 12.9; *n* = 70; *p* < 0.001; 0.12 µL EO L^−1^ air: *χ*^2^ = 21.4; *n* = 71; *p* < 0.001; 0.62 µL EO L^−1^ air*: χ*^2^ = 10.0; *n* = 73; *p* = 0.002; 1.25 µL EO L^−1^ air*: χ*^2^ = 5.7; *n* = 93; *p* < 0.017). On the contrary, the *A. citrodora* EO showed a clear negative chemotaxis with a significant repellent effect at 1.25 and 2.50 µL EO L^−1^ air (1.25 µL EO L^−1^ air*: χ*^2^ = 9.0; *n* = 25; *p* < 0.003; 2.50 µL EO L^−1^ air*: χ*^2^ = 11.6; *n* = 25; *p* = 0.001). Surprisingly, the *A. sativum* EO did not significantly affect the behaviour of *C. vomitoria*, except for the significant repellent effect (*χ*^2^ = 7.0; *n* = 48; *p* = 0.008) at the highest concentration (2.50 µL EO L^−1^ air) (Figure 3).

### 3.4. Toxicity of the Essential Oils on C. vomitoria

The ovicidal bioassays showed that all three EOs are toxic to the eggs of *C. vomitoria*. LC_50_ values ranged from 0.024 to 0.037 μL EO cm^−2^ for the *B. graveolens* and *A. sativum* EOs, respectively (Table 2), with no significant difference in toxicity among the EOs according to the rmp analysis (Table 3).

The toxicity of the three EOs against *C. vomitoria* adult flies was tested by fumigation, contact, and ingestion. By fumigation, LC_50_ values ranged from 1.86 to 25.30 for the *A. sativum* and *B. graveolens* EOs, respectively. The toxicity of the *A. sativum* EO was about ten-fold higher than the toxicity of the two EOs extracted from the Andean plants (Table 4). Consistently, the rmp analysis indicated a significant difference between the *A. sativum* EO and the other two tested EOs, while no significant differences were observed between the EOs from *A. citrodora* and *B. graveolens* (Table 5).

By contact, the *A. citrodora* EO was about four times as toxic as the *B. graveolens* EO and twice as toxic as the *A. sativum* EO, with LD_50_ values ranging from 0.27 to 0.96 μL EO fly^−1^ for *A. citrodora* and *B. graveolens,* respectively (Table 4). According to the rmp analysis, the toxicity of the *A. citrodora* EO was significantly higher than that of *A. sativum* and *B. graveolens* EOs, while the *A. sativum* EO was significantly more toxic than the *B. graveolens* EO.

By ingestion, LC_50_ values ranged from 8.10 and 44.97 μL EO mL^−1^ gel for the *A. sativum* and *B. graveolens* EOs, respectively (Table 4).

Rmp analysis showed that both the *A. citrodora* and *B. graveolens* EOs were significantly less toxic than the *A. sativum* EO, while no significant difference was detected between the *A. citrodora* and *B. graveolens* EOs (Table 5).

## 4. Discussion

The tropical Andes are a biodiversity hotspot, rich in aromatic plant species whose potential as sources of active compounds for the control of insects is still largely underexploited. Here, the EOs extracted from *A. citrodora* and *B. graveolens*, two aromatic plants from the Ecuadorian Andes, were analysed from a chemical and sensory point of view, and their bioactivity against the blue blowfly *C. vomitoria*, a vector of human pathogens and a pest in meat and fish factories and stores, was tested.

The chemical analyses showed that the composition of the EOs is extremely complex. The composition of the *A. citrodora* EO includes geranial and neral as its main compounds, followed by limonene, and it is similar to the leaf volatile oils detected in plants growing in Chile, Argentina, and Jordan [48,49]. The *B. graveolens* EO was strongly characterised by limonene, which accounted for over 45% of the total composition, as already reported for specimens from Ecuador [50,51] and Cuba [52,53].

In the three tested EOs, the chemical composition and the odour profiles matched quite well. The very low pleasantness attributed to the odour of the *A. sativum* EO can be easily explained by the off-flavours detected by the panellists. These off-flavours can be related to the presence of diallyl-sulfide, -disulfide, -trisulfide, -tetrasulfide, methyl allyl trisulfide, and S-propylpropane thiosulfonate, whoseodour is characterised as pungent, sulphurous, onion-garlic-like, and horseradish-like with a metallic nuance. On the contrary, the olfactory expression of the *A. citrodora* EO was described as a sweet, pleasant fragrance with citrus nuances, both fruity and floral. Such perceptions can be attributed to the presence of limonene, neral, geranial, geranyl acetate, and β-caryophyllene. The presence of α-terpineol, myrcene, *cis-* and *trans-* carveol, and menthofuran in the composition of the *B. graveolens* EO perfectly matches its odour, mainly described as fresh and vegetal (mint, menthol), but with a high percentage of mouldy lemon nuances that reduced its pleasantness.

In line with the different chemical composition, a different sensory perception of the EOs was also observed in *C. vomitoria*. According to the behavioural tests, the two EOs distilled from Andean plants showed a clearly different effect on the *C. vomitoria* adults. At the tested concentrations (0.06–2.50 μL EO L^−1^ air), we observed mainly negative chemotaxis of *C. vomitoria* to the *A. citrodora* EO. On the contrary, the *B. graveolens* EO was significantly attractive for the flies, except for at its highest concentration (2.50 μL EO L^−1^ air) which was repellent. The repellence shown by the *A. citrodora* EO was stronger even than the repellence of the *A. sativum* EO that, in this experiment, represented the positive control, since it proved to be effective in discouraging the blowflies from entering a meat processing room of a dry-curing ham factory when sprayed as a mist to build an olfactory barrier [27]. These results indicate that odours may be very differently perceived by humans and insects. The *A. citrodora* EO, judged as pleasant smelling in the sensory analysis and when commonly used by people [34,35], in our study was more repellent to the blowfly *C. vomitoria* than garlic, associated by the human senses with sulphurous and smoky smells. The *B. graveolens* EO, overall pleasant for the panellists but carrying some negative off-flavours, was, instead, clearly attractive to *C. vomitoria*.

Regardless of the behavioural results, the toxicity bioassays performed in this study showed that not only the repellent *A. citrodora*, but also the attractive *B. graveolens* EOs have a clear dose-dependent toxic activity against eggs and adults (by contact, fumigation, and ingestion) of *C vomitoria.*

Both the *A. citrodora* and *B. graveolens* EOs were very effective in preventing the *C. vomitoria* eggs from hatching (100.00 ± 0.67% and 87.06 ± 2.34% eggs’ mortality, respectively, at 0.06 µL EO cm^−2^). Similarly, hatching was almost completely inhibited (99.33 ± 0.67% eggs’ mortality) by the garlic EO, starting from the concentration of 0.16 µL EO cm^−2^. In line with our findings, two *Origanum vulgare* L. (Lamiaceae) EOs, extracted from a carvacrol- and a thymol- chemotype, almost completely prevented *C. vomitoria* eggs from hatching (eggs’ mortality more than 90%), starting from just 0.05 µL EO cm^−2^ [28]. A lower toxicity was instead reported for the EOs extracted from *Salvia officinalis* L. and *Rosmarinus officinalis* L. (Lamiaceae), which showed a reduced hatching only by about 12–20% at a concentration of 0.40 µL EO cm^−2^ [27]. The *A. citrodora* EO was also tested on the eggs of the soybean pest *Nezara viridula* (L.) (Hemiptera: Pentatomidae) [54]. In that study, Werdin González et al. observed that the EO completely inhibited hatching (100% egg’s mortality) at 12.5 μg EO egg^−1^ with an LC_50_ value of 1.9 μg EO egg^−1^.

To the best of our knowledge, no data are available on contact toxicity of the two EOs on adult Diptera. However, in previous studies, the acute toxicity of the *A. citrodora* EO was tested against the larvae of the mosquitoes *Aedes aegypti* L., *Anopheles stephensi* Liston, and *Culex quinquefasciatus* Say (Diptera: Culicidae) in water, with LC_50_ values ranging between about 10–100 ppm [55,56]. Similarly, Leyva et al. [57] recently tested the larvicidal effect of a *B. graveolens* EO, extracted from leaves, on *Ae. aegypti*, *Aedes albopictus* (Skuse) (Diptera: Culicidae)*,* and *C. quinquefasciatus.* They found an LC_50_ of 32.5, 31.8, and 31.5 mg EO L^−1^ of water after 24 h. The toxicity by contact of the *A. citrodora* EO (LD_50_ = 13.8 μg EO insect^−1^ after 72 h) was also demonstrated against adults of the stored-product pest *Tribolium castaneum* Herbst (Coleoptera: Tenebrionidae) by Benzi et al. [58].

The susceptibility of *C. vomitoria* adults to other EOs was previously tested for EOs distilled from aromatic plants traditionally used as culinary herbs [16,25,27,28]. In our study, the strongest toxic effect by contact was that of the *A. citrodora* EO, with an LD_50_ of 0.27 µL EO fly^−1^. Its toxicity was about twice as high as that calculated for the *A. sativum* EO (LC_50_ = 0.46 µL EO fly^−1^) and *Artemisia dracunculus* L. (Asteraceae) (LC_50_ = 0.485 µL EO fly^−1^) EO [25], but similar to that measured for an oregano carvacrol-chemotype EO (LC_50_ = 0.240 µL EO fly^−1^) [28].

By fumigation, the toxic effect against adult blowflies of the *A. sativum* EO (1.86 μL EO L^−1^ air) was more than ten-fold higher than that of the two EOs from the Andean plants (LC_50_ = 23.66 and 25.30 μL EO L^−1^ air for the *A.* *citrodora* and *B. graveolens* EOs, respectively) tested in this study as well as that previously measured for sage and rosemary EOs (LC_50_ = 25.52 and 31.52 µL EO L^−1^ air, respectively) [27]. The *A. sativum* EO toxic effect by fumigation was also much higher than that of the *A. dracunculus* and *Artemisia annua* L. (Asteraceae) (LC_50_ = 49.55 and 88.09 µL EO L^−1^ air, respectively) EOs [25]. As for other insect species, the toxicity of the *A. citrodora* EO by fumigation against *C. vomitoria* found in this experiment (LC_50_ = 23.66 μL EO L^−1^ air) was lower than that against *Callosobruchus maculatus* (Fabricius) (Coleoptera: Chrysomelidae) (LC_50_ = 10.17 μL EO L^−1^ air), but higher than that observed by Khani et al. [59] against *Tribolium confusum* du Val (Coleoptera: Tenebrionidae) (LC_50_ = 497.83 μL EO L^−1^ air).

Both the *A. citrodora* and *B. graveolens* EOs were also toxic to *C. vomitoria* by ingestion. According to our experiment, the two EOs extracted from Andean plants, even if much less toxic than the *A. sativum* EO, managed to kill half of the fly population at concentrations of up to about 40 μL EO mL^−1^ gel after 48 h. Although no data are available about the oral toxicity of EOs for blowflies, a previous study by Buentello-Wong et al. [60] on fruit flies showed that the EOs distilled from *Eugenia caryophyllus* (Spreng.) Bullock and S.G. Harrison (Myrtaceae), *Ocimum basilicum* L., and *Thymus vulgaris* L. (Lamiaceae) were toxic by ingestion to *Anastrepha ludens* (Loew) (Diptera: Tephritidae) with a mortality rate of over 50% at a concentration of 1.5% (*w/v*) after 5 days. Similar to our results, a very variable effect of several EOs was found by Benelli et al. [61] against *Ceratitis capitata* (Wiedemann) (Diptera: Tephritidae) with LD_50_ values ranging from 13 ppm for the *Hyptis suaveolens* (L.) Poit EO to 6870 ppm for the *Lavandula angustifolia* Mill. (Lamiaceae) EO. The observed high effectiveness of the *A. sativum* EO, both by fumigation and ingestion, indicates that it could be an excellent candidate as an active ingredient for botanical-based insecticides. However, its unpleasant smell represents a strong limitation in its practical use.

## 5. Conclusions

Our assays found stronger repellent and toxic (by contact) effects for the *A. citrodora* EO against adults of *C. vomitoria* than those of the *A. sativum,* which was, on the contrary, the most toxic EO by ingestion and fumigation. Therefore, using the *A. citrodora* EO as an active ingredient in a repellent mist spray might be an effective alternative to the previously tested *A. sativum* to control *C. vomitoria* in houses and stores, because of its pleasant lemony scent. Due to its attractiveness, the *B. graveolens* EO could be, instead, used in bait traps to lure and kill *C. vomitoria*. Even if repellent and insecticidal properties were demonstrated in a large number of EOs, our results suggest that, when selecting the right EO, it is crucial to consider not only its effects on the target pest species, but also its impact on the human senses and its suitability for different purposes (e.g., monitoring, lure and kill traps, mist dispensers, and topical formulations).

## Figures and Tables

**Table 1:** Chemical compositions of *Allium sativum*, *Aloysia citrodora*, and *Bursera graveolens* essential oils (EOs).

Compound	l.r.i. ^a^	Relative Abundance (%)		
		*Allium sativum* *	*Aloysia citrodora*	*Bursera graveolens*
diallyl sulfide	866	5.5	*-* ^b^	*-*
2,3-dimethyl thiophene	901	0.3	*-*	*-*
methyl-2-propenyl disulfide	920	3.6	*-*	*-*
(*Z*)-methylpropenyl disulfide	932	0.2	*-*	*-*
(*E*)-methylpropenyl disulfide	940	0.2	*-*	*-*
α-pinene	941	-	0.3	-
dimethyl trisulfide	975	0.8	-	-
sabinene	978	*-*	0.9	-
1-octen-3-ol	980	*-*	0.2	-
6-methyl-5-hepten-2-one	986	*-*	0.6	-
myrcene	991	*-*	0.2	3.0
3- octanol	994	*-*	0.1	-
p-cymene	1028	*-*	-	1.1
limonene	1032	*-*	7.2	46.2
1,8- cineole	1033	*-*	2.2	-
(*Z*)-β-ocimene	1042	*-*	0.1	-
(*E*)-β-ocimene	1052	*-*	2.4	-
*cis*-sabinene hydrate	1070	-	0.2	-
diallyl disulfide	1082	16.1	-	-
linalool	1101	-	0.2	-
(*E*)-1-allyl-2-(prop-1-en-1-yl) disulfane	1103	0.7	-	-
(*Z*)-1-allyl-2-(prop-1-en-1-yl) disulfane	1107	0.6	-	-
*trans*-*p*-mentha-2,8-dien-1-ol	1121	-	-	0.5
*cis*-*p*-mentha-2,8-dien-1-ol	1135	-	-	0.4
*trans*-limonene oxide	1139	-	-	0.5
methyl allyl trisulfide	1142	9.5	-	-
β-terpineol	1153	-	-	0.4
menthone	1148	-	-	1.0
4-methyl-1,2,3-trithiolane	1154	0.9	-	-
β-pinene oxide	1155	-	0.6	-
citronellal	1156	-	0.2	-
menthofurane	1165	-	-	3.4
*iso*neral	1170	-	0.6	-
*iso*geranial	1184	-	0.9	-
α-terpineol	1190	-	0.6	17.8
*cis*-dihydrocarvone	1194	-	-	0.7
*cis*-piperitol	1195	-	-	0.7
2-vinyl-4H-1,3-dithiine	1206	0.6	-	-
dimethyl tetrasulfide	1210	0.8	-	-
*trans*-carveol	1220	-	-	2.1
*cis*-carveol	1228	-	-	5.0
nerol	1230	-	0.6	-
pulegone	1239	-	-	0.8
neral	1240	-	21.0	-
carvone	1244	-	-	1.3
geraniol	1257	-	0.4	-
geranial	1271	-	26.8	-
diallyl trisulfide	1297	23.1	-	-
(*Z*)-1-allyl-3-(prop-1-en-1-yl) trisulfane	1329	0.2	-	-
(*E*)-1-allyl-3-(prop-1-en-1-yl) trisulfane	1346	0.6	-	-
S-methyl-1,2,3,4-tetrathiane	1364	1.0	-	-
α-copaene	1377	-	0.4	-
geranyl acetate	1385	-	2.4	-
S-propylpropane thiosulfonate	1388	6.7	-	-
α-cedrene	1409	-	0.2	-
β-caryophyllene	1419	-	2.7	-
1-(1-(methylthio)propyl)-2-propyl disulfane	1431	0.5	-	-
dimethyl pentasulfide	1450	0.3	-	-
α-humulene	1455	-	0.1	-
*allo*aromadendrene	1462	-	0.4	-
γ-muurolene	1477	-	-	0.8
geranyl propionate	1478	-	0.3	-
germancrene D	1482	-	3.1	-
*ar*-curcumene	1483	-	3.1	-
bicyclogermancrene	1496	-	6.8	-
mint lactone	1499	-	-	1.3
β- curcumene	1513	-	1.1	-
cubebol	1515	-	0.8	-
δ- cadinene	1524	-	0.2	-
diallyl tetrasulfide	1540	17.4	-	-
(*E*)-nerolidol	1564	-	2.0	-
spathulenol	1572	-	4.4	0.7
germancrene D-4 ol	1575	-	1.1	-
1-propyl-2-(4-thiohept-2-en-5-yl) disulfide	1580	0.2	-	-
caryophyllene oxide	1581	-	2.0	-
6-methyl-4,5,8-trithia-1,10-undecadiene	1597	0.9	-	-
α-*epi*-7-*epi*-5-eudesmol	1617	-	-	5.0
3-amino-*tert*-butyl benzoate	1620	0.9	-	-
*iso*spathulenol	1639	-	0.5	-
τ-cadinol	1641	-	0.7	0.8
α-cadinol	1653	-	-	1.3
α-bisabolol	1684	-	-	0.7
1-allyl-3-(2-(allylthio)propyl) trisulfane	1818	2.0	-	-
cyclic octaatomic sulfur	2030	0.2	-	-
1-allyl-3-(2-(allyldisulfanyl)propyl) trisulfane	2066	1.2	-	-
Monoterpene hydrocarbons		-	11.1	50.3
Oxygenated monoterpenes		-	57.0	35.4
Sesquiterpene hydrocarbons		-	18.1	0.8
Oxygenated sesquiterpenes		-	11.5	9.2
Nitrogen compounds		0.9	-	-
Sulfur compounds		94.0	-	-
Other non-terpene derivatives		-	0.9	-
Total identified (%)		94.8	98.6	95.7

^a^ Linear retention index on a DB-5 capillary column; ^b^ Not detected; * Data from Bedini et al. [27].

**Table 2:** Toxicity of *Aloysia citrodora, Bursera graveolens*, and *Allium sativum* essential oils (EOs) to eggs of the blowfly *Calliphora vomitoria*.

EO	LC_50_ (95% FL)	Intercept ± SE	*p*
*A. citrodora*	0.034 (0.024–0.049)	3.811 ± 0.139	<0.001
*B. graveolens*	0.024 (0.017–0.034)	4.185 ± 0.145	<0.001
*A. sativum*	0.037 (0.022–0.060)	3.722 ± 0.131	<0.001

Data are given as μL EO cm^−2^. FL, fiducial limits. Model slope = 2.595 ± 0.088; Pearson goodness-of-fit test, *χ*^2^ = 244.094, df = 14, *p* < 0.001.

**Table 3:** Relative toxicity of *Aloysia citrodora*, *Bursera graveolens*, and *Allium sativum* essential oils (EOs) to eggs of the blowfly *Calliphora vomitoria*.

	EO (X)	*A. citrodora*	*B. graveolens*
EO (Y)			
*B. graveolens*		1.394 (0.876–2.458)	-
*A. sativum*		0.924 (0.465–1.662)	0.663 (0.300–1.196)

Rmp estimates for paired comparisons of the LC_50_ values. In brackets, 95% confidence intervals. Values < 1 and >1 indicate higher and lower toxicity, respectively, of the compared EOs.

**Table 4:** Toxicity by fumigation, contact, and ingestion of *Aloysia citrodora*, *Bursera graveolens*, and *Allium sativum* essential oils (EOs) to adults of the blowfly *Calliphora vomitoria*.

EO	LC_50_/LD_50_ (95% FL)	Intercept ± SE	*p*
**Fumigation**			
*A. citrodora*	23.657 (18.698–30.706)	−4.161 ± 0.369	<0.001
*B. graveolens*	25.303 (19.975–33.190)	−4.250 ± 0.372	<0.001
*A. sativum*	1.860 (1.250–2.760)	−0.816 ± 0.161	<0.001
**Contact**			
*A. citrodora*	0.268 (0.189–0.367)	1.168 ± 0.148	<0.001
*B. graveolens*	0.958 (0.712–1.430)	0.038 ± 0.107	0.723
*A. sativum*	0.462 (0.291–0.750)	0.686 ± 0.167	<0.001
**Ingestion**			
*A. citrodora*	35.645 (23.449–52.870)	−4.068 ± 0.402	<0.001
*B. graveolens*	44.975 (30.019–68.448)	−4.333 ± 0.412	<0.001
*A. sativum*	8.094 (5.322–12.182)	−2.380 ± 0.252	<0.001

LC_50_/LD_50_, concentration/dose of EO that kills 50% of the specimens. Fumigation: data given as μL EO L^−1^ air; model slope = 3.029 ± 0.271; Pearson goodness-of-fit test, *χ*^2^ = 33.286, df = 13, *p* = 0.002. Contact: data given as μL EO fly^−1^; model slope = 2.043 ± 0.222; Pearson goodness-of-fit test, *χ*^2^ = 21.593 df = 13, *p* = 0.062. Ingestion: data given as μL EO mL^−1^ gel; model slope = 2.621 ± 0.243; Pearson goodness-of-fit test, *χ*^2^ = 33.567, df = 11, *p* < 0.001.

**Table 5:** Relative toxicity by fumigation, contact, and ingestion of *Aloysia citrodora*, *Bursera graveolens*, and *Allium sativum* essential oils (EOs) to adults of the blowfly *Calliphora vomitoria*.

	EO (X)	*A. citrodora*	*B. graveolens*
EO (Y)			
**Fumigation**			
*B. graveolens*		0.935 (0.653–1.313)	-
*A. sativum*		**12.721 (3.703–124.904)**	**13.606 (3.880–139.0.38)**
**Contact**			
*B. graveolens*		**0.280 (0.108–0.498)**	-
*A. sativum*		**0.581 (0.286–1.008)**	**2.076 (1.189–4.503)**
**Ingestion**			
*B. graveolens*		0.793 (0.418–1.367)	-
*A. sativum*		**4.404 (1.753–26.592)**	**5.557 (2.050–39.806)**

Rmp estimates for paired comparisons of the LC_50_ values for the tested EOs, with 95% confidence intervals in brackets; the values < 1 and >1 indicate higher and lower toxicity, respectively, of the compared EOs. The bolded values indicate significant differences.

## Data Availability

The data presented in this study are available on request from the corresponding author.
